# On the Use of Warm and Hot Water in Surgery

**Published:** 1875-08

**Authors:** Frank H. Hamilton

**Affiliations:** Surgeon to Bellevue Hospital, New York


### Selections.

### ON THE USE OF WARM AND HOT WATER IN SURGERY.

By FRANK H. HAMILTON,

SURGEON TO BELLEVUE HOSPITAL, NEW YORK.

Gentlemen: It is many years since I had an opportunity of addressing you as members of this Society. In the meantime experience has brought to me many new and interesting observations. From these I have selected that one, as the theme of a few remarks, in which my interest has been the most recent, and, perhaps, I might justly say, the most profound, namely, the use of *Warm and Hot Water in Surgery.*

In 1851, Amussat, of Paris, sent me a copy of his monograph on the "Use of Water in Surgery," which I translated and published in the *Buffalo Medical Journal.* In this brochure the conclusion was reached that, as a rule, that temperature was most useful which was found on trial to be the most agreeable; and that, in most cases, this would be a temperature of about 68°, Fahrenheit; that is to say, water at a temperature considerably below the natural temperature of the body, but not cold water. During most of my life I have been guided by the experience of Amussat, and have employed water, as an external application in surgery, at a temperature which might be called tepid or cool. Two years ago I was made consulting and operating surgeon to the St. Francis Hospital, in New York City—a hospital of two hundred and fifty or three hundred beds—and found the gentlemen in that institution, all thoroughly educated German physicians, using warm and hot water in nearly all cases of surgical accidents. Dr. Achilles Rose, who had the principal care of the surgical accidents, was more especially interested in the subject, and first called my attention to it. The practice was new to me, and had been imported, I was informed, from the practice of certain German surgeons. I watched with care the results, and I soon became convinced that here was a means of controlling inflammation, and of preventing the numerous accidents incident to traumatic inflammation, of most wonderful efficacy.

To be brief, during two years or more of service in that hospital, I have never seen a case of gangrene extending beyond the parts orginally injured, nor of erysipelas originating during the treatment, nor of pyæmia or septicæmia. I have also employed the same method for two years, when, as surgeon-in-chief, I was in charge of the Reception Hospitals, and the same practice is now employed in the wards under my care at the Bellevue Hospital. My experience, therefore, includes the management and observation of probably more than one hundred cases, with either hot or warm water, and all has tended to confirm my early convictions of the value of this plan. It cannot be denied that both cool and cold water are sometimes effective in preventing, or in arresting, the progress of inflammation; but it is plain to me that warm and hot water do this more certainly, and without the danger of those terrible accidents, such as gangrene, caused by the depression of the temperature and consequent devitalization of the parts.

There are two modes of using the water: one by submersion in a bath, and the other by fomentation. Of these the bath is the best; but it can only be employed in cases of injuries of the hand and forearm, or of the foot and leg. Those which we employ are manufactured by Otto and Rynders, and are made of zinc, and are furnished with stop-cocks for the purpose of renewing the water at pleasure. The temperature employed for ordinary injuries is from 95° to 100°, Fahrenheit, but in cases of gangrene we elevate the temperature to 105° or 110° sometimes. Slight variations in temperature do not affect the results, and in most cases it is found sufficient to change the water three times daily. It will be understood that neither sutures, adhesive plasters or rollers are employed. Union by "first intention" is not therefore expected by this method. If we desire primary union we must employ fomentations, not submersion; nor even then shall we avoid secondary accidents so certainly as when we omit sutures and bandages.

When a limb is submerged it soon becomes œdematous, partly in consequence of the depending position and partly in consequence of endosmosis; and this œdema is so great often as to cause apprehension in the minds of those inexperienced in its use; but it will be found almost always that there is no tenderness on pressure, and if the limb is removed from the bath at the end of twelve or fourteen days the œdema will soon disappear. Let me not omit to say, that in order to avoid all danger of a hæmorrhage, we rarely submerge a limb until twelve or twenty hours after the accident.

Fomentations are employed after the lapse of twelve or fourteen days in nearly all cases, and occasionally at night when the patient is weary of the bath; and in other cases in which we cannot use the bath, fomentations are employed from the beginning.

If *fomentations* are employed, the parts must be kept *thoroughly saturated* with water. Merely moistening the surface will not accomplish the results desired. Three or four sheets of lint should be soaked in the water and wrapped about the injured parts, and over this a large mass of cotton batting should be placed, the whole being enclosed in soft vulcanized rubber or oil silk. This may require to be renewed three or four times daily.

Permit me to relate one or two cases by way of illustration. A man was brought to the Park Reception Hospital whose foot had been traversed by the wheel of a car, crushing the bones and the flesh below the tarsus. Every one knows how certainly such an accident proves fatal if we do not amputate the foot, and we can seldom amputate below the ankle joint. This limb was immediately placed in a bath at a temperature of about 95° or 100° Fahrenheit. During the following six or seven days he suffered very little pain; there was no traumatic fever, nor did the gangrene extend a line beyond the parts originally injured. About this time, at one of my visits, I discovered that gangrene had commenced above the seat of the injury, and had within a period of twenty-four hours, extended about one inch. It was now ascertained that the temperature of the bath had become very much lowered, so that to my hand it felt cool. The orderly had omitted to renew the water. At once the cool water was withdrawn, and water at a temperature of 105° Fahrenheit substituted. On the following morning the gangrene was arrested, and from this time the wound continued to do well, and a recovery eventually took place, with only the loss of the extremity of his foot.

A man was brought to the 99th Street Reception Hospital with a compound fracture of the tibia and fibula, the fracture being a little below the middle of the leg. The house surgeon attempted to save the limb, but gangrene ensued, and on the third day I was summoned. The gangrene had now reached the middle of the leg, and was extending rapidly upwards, while an erysipelatous inflammation reached above the knee. His pulse was rapid and his general condition very bad. It is scarcely necessary to say, that under these circumstances amputation would probably have hastened a fatal termination. As soon as the bath could be made ready, the limb was submerged in water at a temperature of about 105° Fahrenheit. The following day his general condition had much improved, and on the ninth day, I think it was, the limb was spontaneously separated at the point of fracture. From the hour that the limb was submerged the erysipelas began to decline, and the gangrene was arrested.

I cannot venture to occupy your time with any more extended remarks, as there is so much unfinished business pressing on the Society, but may refer you to the *Richmond and Louisville Medical Journal* (published at Louisville, Ky.) for January, 1874, in which I have given a more full account of the mode of precedure in these cases; also to the *New York Medical Record*, of May 15, 1874, containing a supplement to these observations.

EDITOR'S NOTE.—The above article is reprinted from a small pamphlet just issued by G. P. Putnam's Sons, New York, in order to direct attention to a subject which so well deserves it. The testimony of an expert, like Dr. Hamilton, equally distinguished for his honesty and prudence, as for his sagacity and skill, does not need the ample corroboration supplied in his admirably conducted wards at Bellevue Hospital, where the good results of the practical application of his conclusions are apparent.

The value of this agent in surgical practice, has moreover been thoroughly demonstrated by Prof. Moses Gunn, of Rush Medical College, both in his surgical clinic, and in his wards at St. Joseph's Hospital, and has received his cordial approval. So well is the professor's predilection for getting his patients into actual hot water understood by the students, that they have learned to expect metaphorical hot water for themselves if they neglect it. H.

